# Myocardial injury complicating diabetic ketoacidosis in type 1 diabetes mellitus with concurrent thyroid storm: Case report and literature review

**DOI:** 10.1097/MD.0000000000045596

**Published:** 2025-11-14

**Authors:** Yingjuan Ma, Ruirui Liu, Ting Bai, Xinyue Li, Wang Chang, Limin Jia

**Affiliations:** a People’s Hospital of Ningxia Hui Autonomous Region, Ningxia Medical University, Yinchuan City, Ningxia Hui Autonomous Region, China; b The Third Clinical Medical College of Ningxia Medical University, Yinchuan City, Ningxia Hui Autonomous Region, China.

**Keywords:** comorbidity and coexistence, diabetic ketoacidosis, myocardial injury, thyroid storm, type 1 diabetes

## Abstract

**Rationale:**

Diabetic ketoacidosis (DKA) and thyrotoxic storm are life-threatening endocrine emergencies that sometimes occur together, but myocardial injury in this setting is rare. This case is unique in showing myocardial injury during simultaneous DKA and thyroid storm (TS), highlighting clinical complexity and raising questions about underlying pathophysiological mechanisms. We highlight the rarity of this presentation and explore how DKA and TS may cause myocardial injury through metabolic derangements, electrolyte disturbances, and inflammatory-mediated damage.

**Patient concerns:**

The patient is a 23-year-old male who was admitted primarily due to an 8-year history of intermittent dry mouth, polydipsia, and polyuria, followed by the acute onset of palpitations, fever, and vomiting with blood glucose 25 mmol/L, urine glucose +++, urine ketones +++, arterial blood gas analysis shows pH: 7.190, HCO3−: 7.6 mmol/L, free triiodothyronine: 6.95 pg/mL, free thyroxine: 4.83 ng/dL, thyroid-stimulating hormone < 0.008 uIU/mL. He has a documented medical history of type 1 diabetes mellitus and hyperthyroidism.

**Diagnoses:**

Admission diagnosis: type 1 DKA; TS; electrolyte imbalance; upper respiratory tract infection. Discharge diagnosis: type 1 DKA; TS; electrolyte imbalance; myocardial damage; upper respiratory tract infection.

**Interventions:**

Regarding the treatment strategy, a multidisciplinary collaborative approach was implemented in this case, incorporating the management of endocrine disorders and cardioprotective measures.

**Outcomes:**

Following meticulous management, the patient achieved full recovery from DKA and TS, blood sugar levels dropped and urine ketone bodies disappeared. The thyroid function indicators free triiodothyronine and free thyroxine have shown a decrease compared to previous levels. The blood indicators of myocardial injury have decreased, electrocardiogram has returned to normal. The patient was subsequently discharged from the hospital in stable condition.

**Lessons:**

This case not only illustrates the efficacy of a comprehensive treatment strategy but also reinforces the importance of close patient monitoring, prompt interpretation of clinical data, and early intervention in the context of multiple concurrent endocrine crises. By documenting this rare presentation, the report contributes valuable clinical insights and serves as a reference for the diagnostic and therapeutic management of similar cases, emphasizing the critical role of multidisciplinary collaboration in optimizing outcomes in critical care settings.

## 1. Introduction

Diabetic ketoacidosis (DKA) is a prevalent condition frequently encountered in emergency departments and endocrinology units.^[1]^ Additionally, clinical cases have been documented where DKA coexists with thyroid storm (TS).^[2]^ The comorbidity status not only increases mortality rates and treatment complexity, but also complicates the underlying pathophysiological mechanisms. The concurrent myocardial injury under such intricate conditions has been scarcely documented in the literature. This article presents a case report of myocardial injury in a young male patient with concurrent type 1 DKA and TS. Through a comprehensive literature review, the study further investigates the underlying mechanisms and etiological factors contributing to myocardial injury in this clinical scenario.

DKA is a severe metabolic disorder syndrome characterized by insulin deficiency and inappropriate elevation of counter-regulatory hormones, leading to profound disturbances in carbohydrate, lipid, and protein metabolism. The condition is clinically manifested by hyperglycemia, ketonemia, and metabolic acidosis.^[3]^ Patients with type 1 diabetes mellitus (T1DM) exhibit a predisposition to spontaneous DKA, typically manifesting as acute and significant diabetic symptoms accompanied by markedly elevated blood glucose levels. Individuals presenting with DKA and concomitant troponin elevation demonstrate an increased risk of major adverse cardiovascular events.^[4,5]^ The mortality rate associated with DKA ranges from 10% to 20%.^[6]^ TS, also known as thyrotoxic crisis, is characterized by the acute exacerbation and deterioration of hyperthyroidism symptoms, presenting with multisystem involvement. Common precipitating factors include infection, surgery, trauma, and psychological stress.^[7]^ With a mortality rate of 25% to 30%,^[8]^ multiorgan failure constitutes the predominant cause of death, necessitating prompt recognition and emergent therapeutic intervention. Both DKA and TS represent life-threatening medical emergencies that can induce severe myocardial impairment,^[8]^ potentially progressing to cardiogenic shock. When these conditions coexist, the pathophysiological mechanisms become notably complex, thereby increasing therapeutic challenges. The incidence of malignant arrhythmias or multiple organ failure escalates significantly, leading to a marked rise in mortality rates. According to literature reports, the mortality rate in such cases can reach up to 15%.^[9]^ This case involves DKA complicated by TS, during which elevated myocardial injury markers and acute myocardial infarction (AMI)-like changes on electrocardiogram were observed during treatment. Following therapeutic intervention and observation, the condition was ultimately attributed to myocardial injury caused by noncoronary infarction (“pseudomyocardial infarction”). This report presents a comprehensive analysis of the diagnostic and therapeutic approaches employed in this case.

## 2. Patient information

A 23-year-old male patient, employed as an office worker engaged in mental labor, was admitted to the hospital with the chief complaints of “intermittent dry mouth, polydipsia, and polyuria for 8 years, accompanied by palpitations, fever, and vomiting for 1 day.” The patient was diagnosed with T1DM at the age of 15 and has presented to the emergency department multiple times for DKA. Currently, the patient is undergoing basal-bolus insulin therapy for glycemic control, which has been suboptimal. One day prior to admission, the patient developed cough and fever without sputum or palpitations, accompanied by nausea and vomiting of gastric contents, sore throat, and diarrhea (watery stool, 5–6 times per day). No precordial pain was reported. Initial evaluation at a local hospital revealed positive Mycoplasma pneumoniae IgM, positive fecal occult blood, and presence of pus cells. Despite symptomatic treatment, the patient’s condition showed no significant improvement. On February 11, 2025, the patient was admitted to our hospital with the following vital signs: heart rate 170 beats/min, respiratory rate 30 breaths/min, blood pressure 120/60 mm Hg, and body temperature 38 °C. Physical examination revealed a normal-built individual (height 173 cm, weight 58 kg) with poor mental status but clear consciousness, and congested posterior pharyngeal wall. Bilateral tonsils show no significant erythema or swelling. The thyroid gland is not visibly enlarged, with a soft consistency. Fine tremors are present in both hands. Heart rate is 170 beats per minute with a regular rhythm, and no significant pathological murmurs are auscultated. Breath sounds are coarse in both lungs. The abdomen is soft with mild tenderness under the xiphoid process, without rebound tenderness. The remainder of the abdomen is soft and nonresistant. Muscle strength is grade 5 in all extremities with normal muscle tone. No edema is observed in the lower extremities. Random blood glucose is 25 mmol/L, urine glucose +++, urine ketones +++, arterial blood gas analysis shows pH 7.190, HCO3− 7.6 mmol/L. Fasting C-peptide is 0.06 ng/mL, 2-hour postprandial C-peptide is 0.07 ng/mL, and HbA1c is 10.4%. The patient has a 5-year history of hyperthyroidism, previously treated with oral methimazole, self-discontinued for 2 years without regular follow-up. Additional auxiliary examinations are detailed in Table 1.

**Table 1:** Primary laboratory tests conducted during patient hospitalization.

Test name Hospitalization	January 11, 2025 Day 1	January 12, 2025 Day 2	January 13, 2025 Day 3	January 14, 2025 Day 4	January 15, 2025 Day 5	January 16, 2025 Day 6	January 17, 2025 Day 7	January 18, 2025 Day 8	Normal reference
*Blood test*									
WBC	17.29	9.2	7.43	–	3.8	–	–	–	3.5–9.5 × 10^9^/L
NEU#	15.21	7.45	6.53	–	2.27	–	–	–	1.8–6.3 × 10^9^/L
NEUT%	88	81	87.8	–	59.6	–	–	–	40–75%
Hb	159	129	137	–	137	–	–	–	130–175 g/L
RBC	5.48	4.39	4.68	–	4.71	–	–	–	4.3–5.8 × 10^12^/L
HCV	48.4	37.6	40.5		39.9				40–50%
PLT	293	175	158	–	172	–	–	–	125–350 × 10^9^/L
HCT	48.4	37.6	40.5	–	39.9	–	–	–	40–50%
MYO	–	–	<21	<21	37.64	88.87	25.02	42.98	28–72 ng/mL
hs TnI	21	–	–	3686.7	-	–	–	67.7	Female ≤ 15.60 pg/mL; male ≤ 34.20 pg/L

Test name Hospitalization	January 11, 2025 Day 1	January 12, 2025 Day 2	January 13, 2025 Day 3	January 14, 2025 Day 4	January 15, 2025 Day 5	January 16, 2025 Day 6	January 17, 2025 Day 7	January 18, 2025 Day 8	Normal reference
hs TnT	–	–	0.9420	0.8100	0.6580	0.4970	0.2090	0.0770	0–0.018 ng/mL
NT-proBNP	876	–	1185	880.9	595	–	–	–	0–125 pg/mL
PH	7.19	–	7.344	–	–	–	–	–	7.35–7.45
PaO_2_	97.9	–	89.8	–	–	–	–	–	83–108 mm HG
PaCO_2_	20	–	31.1	–	–	–	–	–	35–45 mm HG
Lac	1.8	–	1.9	–	–	–	–	–	0.5–1.6 mmol/L
HCO_3_−	12.1		16.9						22–26 mmol/L
AB	7.6	–	16.9	–	–	–	–	–	22–26 mmol/L
SBE	-20.6	–	−8.8	–	–	–	–	–	−3–3 mmol/L
BE	-18.3	–	−7.6	–	–	–	–	–	−3–3 mmol/L
FPG	–	7.6	–	10.6	9.5	7.2	8.8	7.9	3.9–6.1 mmol/L
FT3	6.95	–	–	4.04	–	–	–	3.91	2.3–4.2 pg/mL

Test name Hospitalization	January 11, 2025 Day 1	January 12, 2025 Day 2	January 13, 2025 Day 3	January 14, 2025 Day 4	January 15, 2025 Day 5	January 16, 2025 Day 6	January 17, 2025 Day 7	January 18, 2025 Day 8	Normal reference
FT4	4.83	–	–	3.07	–	–	–	2.48	0.89–1.76 ng/dL
TSH	<0.008	–	–	<0.008	–	–	–	<0.008	0.55–4.78 uIU/mL
TGAb	–	–	–	<0.9	–	–	–	–	0–4.5 IU/mL
TPOAb	–	–	–	>1300.0	–	–	–	–	0–60 U/mL
A-TSHR	–	–	–	4.51	–	–	–	–	0–1.75 IU/L
CREA	86.6	37	34	–	–	–	–	–	41–73 umol/L
BUN	12.78	5.8	6.3	–	–	–	–	–	2.6–7.5 umol/L
UA	852	425	377	–	–	–	–	–	155–357 umol/L
ALB	41.9	–	33.1	–	32.5	–	–	–	40–55 g/L
ALT	19.1	–	28.4	–	16.8	–	–	–	9–50 U/L
AST	15.6	–	29.8	–	8.9	–	–	–	15–40 U/L

Test name Hospitalization	January 11, 2025 Day 1	January 12, 2025 Day 2	January 13, 2025 Day 3	January 14, 2025 Day 4	January 15, 2025 Day 5	January 16, 2025 Day 6	January 17, 2025 Day 7	January 18, 2025 Day 8	Normal reference
K	4.66	3.6	3.76	–	3.1	3.66	–	–	3.5–5.3 mmol/L
Na	130	139	137	–	144	144	–	–	137–147 mmol/L
CL	95	110	102	–	105	104	–	–	99–110 mmol/L
GS-CRP	31.91	12.04	6	–	<6.00	–	–	–	0–6 mg/L
PCT	–	1.2	0.549	–	0.123	–	–	–	0–0.046 ng/mL
HbA1c	–	10.4	–	–	–	–	–	–	4.0–6.0%
*Urine test*									
PRO	1+	–	Negative	Negative	Negative	–	Negative	–	Negative
KET	3+	–	3+	2+	+-	–	Negative	–	Negative
GLU	4+	–	4+	4+	4+	–	±	–	Negative
24 h I (mL)	4032	5240	5180	4302	3988	–	–	–	–
24 h O (mL)	1000	4400	5500	4400	4050	–	–	–	–

24-h I = 24-hour fluid intake, 24-h O = 24-hour fluid output, AB = actual bicarbonate, ALB = albumin, ALT = alanine aminotransferase, AST = aspartate aminotransferase, A-TSHR = thyroid-stimulating hormone receptor antibody, BE = base excess of whole blood, BUN = blood urea nitrogen, CL = chloride, CREA = creatinine, FPG = fasting plasma glucose, FT3 = free triiodothyronine, FT4 = free thyroxine, GLU = urine glucose, GS-CRP = high -sensitive C-reactive protein, Hb = hemoglobin, HbA1c = glycated hemoglobin A1c, HCT = hematocrit, hs TnI = high-sensitivity troponin I, K = potassium, KET = urine ketone bodies, Lac = lactic acid, MYO = myoglobin, NA = sodium, NEU# = neutrophil count, NEUT% = neutrophil percentage, NT-proBNP = N-terminal pro-B-type natriureticpeptide, PaCO_2_ = partial pressure of carbon dioxide in arterial blood, PaO_2_ = partial pressure of oxygen in arterial blood, PCT = procalcitonin, PH = blood pH, PLT = platelet, PRO = urine protein, RBC = red blood cell, SBE = standard base excess, TGAb = antithyroglobulinantibody, TNT-HS = high-sensitivity troponin T, TPOAb = antithyroid peroxidase antibody, TSH = thyroid-stimulating hormone, UA = uric acid, WBC = white blood cell count.

## 3. Diagnostic assessment

The patient, an adolescent male with a prior diagnosis of T1DM, has presented to the emergency department multiple times for DKA. On this occasion, he presented with cough, fever, and palpitations. Based on his medical history, blood glucose levels, arterial blood gas analysis, and the presence of glycosuria and ketonuria, a diagnosis of type 1 DKA was confirmed. The patient was diagnosed with hyperthyroidism 5 years ago and was previously treated with oral methimazole. After discontinuing medication for 2 years, current laboratory findings reveal elevated free triiodothyronine (FT3) and free thyroxine levels, decreased thyroid-stimulating hormone, and elevated TSAb. Thyroid ultrasound indicates diffuse thyroid disease. The diagnosis of hyperthyroidism is unequivocally established. Based on the BWPS scoring criteria for TS,^[10]^ the patient scored 55 points (elevated thyroid hormone levels + elevated body temperature: 10 points, tachycardia: 25 points, moderate gastrointestinal symptoms: 10 points, with precipitating factors: 10 points). According to the JTA diagnostic criteria for TS,^[11]^ which include elevated thyroid hormone levels combined with fever, tachycardia, and gastrointestinal symptoms, the patient was diagnosed with TS.

Admission diagnosis: type 1 DKA; TS; electrolyte imbalance; upper respiratory tract infection.

## 4. Therapeutic intervention

**Figure 1.**
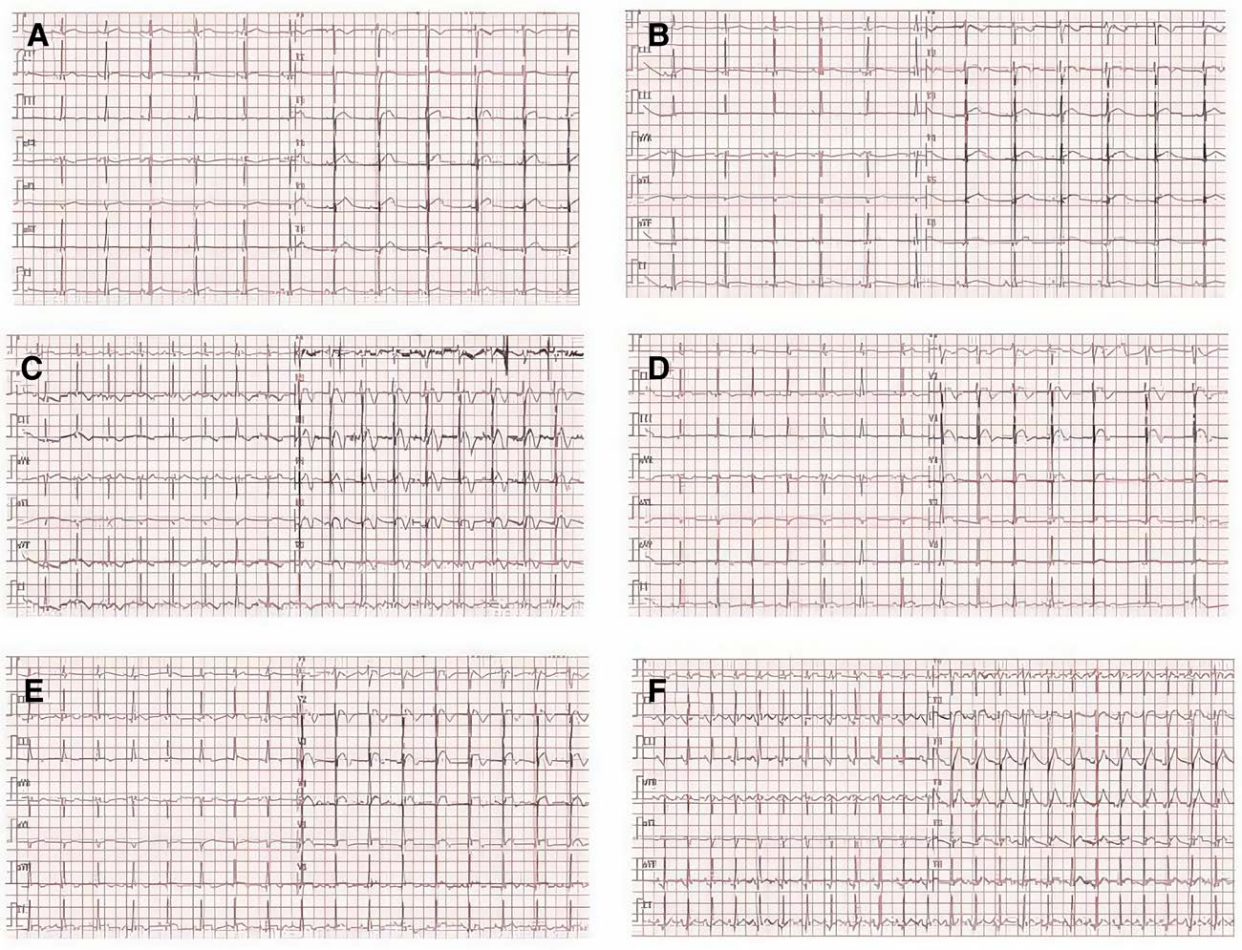
Dynamic electrocardiographic changes during patient treatment. (A) January 11, 2025 16:03:03 (Day 1). (B) January 13, 2025 15:46:05 (Day 3). (C) January 13, 2025 17:08:24 (Day 3). (D) January 14, 2025 07:45:12 (Day 4). (E) January 16, 2025 11:27:52 (Day 6). (F) January 18, 2025 11:42:07 (Day 8).

*For type 1 DKA*: upon hospital admission, in accordance with the latest Chinese Diabetes Prevention and Treatment Guidelines,^[1]^ the patient received comprehensive treatment including causative factor correction, anti-inflammatory therapy, fluid replacement, glucose-lowering therapy, and correction of electrolyte and acid–base imbalances, and Chinese herb Lanqin Oral liquid were administered. Within 2 days of hospitalization, the patient’s body temperature stabilized at 37.5 °C, with improvements in cough, palpitations, nausea, and reduced bowel movement frequency. Pharyngeal pain showed significant relief, and posterior pharyngeal wall congestion improved. Continuous intravenous insulin infusion at a low dose (3–6 units/h) was administered for glycemic control. To address dehydration, a fluid replacement volume of 6000 mL was administered within the first 24 hours, resulting in the resolution of ketoacidosis. For the management of TS, administer hydrocortisone 100 mg tid to counteract stress response, methimazole 20 mg tid to inhibit thyroid hormone synthesis and release, and propranolol 10 mg tid to control heart rate. On the third day of hospitalization, the patient’s body temperature normalized with a maximum of 37 °C, and no further complaints of nausea, vomiting, or diarrhea were reported. The heart rate exhibited a gradual decline, with significant improvement in palpitation symptoms. Electrocardiographic monitoring revealed T-wave inversion in lead II. The patient did not present with notable chest tightness, shortness of breath, precordial pain, shoulder or back pain, or dyspnea. Cardiac auscultation indicated a heart rate of 96 beats per minute with sinus rhythm, and no cardiac murmurs or abnormal heart sounds were detected. The hsTnI level increased from 21 pg/L to 3686.7 pg/L (normal range: 0–34.20 pg/L). The electrocardiogram demonstrated ST-segment elevation and T-wave abnormalities in leads II, III, aVF, and V1 to V6 (Fig. 1). Echocardiography showed an ejection fraction of 65.8% with no abnormalities in wall motion. Following consultation with the cardiology department, myocardial injury was considered, and it was recommended to continue treatment of the primary condition while closely monitoring the patient’s clinical status. Subsequent follow-up revealed no recurrence of precordial discomfort in the patient. Dynamic electrocardiogram monitoring and myocardial enzyme markers exhibited progressive changes. With the correction of ketoacidosis and alleviation of hypermetabolic symptoms, the BWPS score decreased to 15 points (tachycardia 5 points, predisposing factors 10 points) on the 4th hospital day. By the 8th hospital day, the patient’s electrocardiogram gradually normalized, accompanied by a progressive decline in troponin levels. Hydrocortisone was tapered and discontinued, while methimazole dosage was adjusted to 10 mg once daily orally. The patient demonstrated stable blood glucose levels and significant clinical improvement, subsequently meeting discharge criteria (Table 1 and Fig. 1).

## 5. Discussion

Based on the existing literature, the mechanisms by which DKA induces myocardial injury are as follows: metabolic stress and imbalance between myocardial oxygen supply and demand: in the state of hyperglycemia, with absolute or relative insulin deficiency, the body experiences impaired glucose utilization and increased lipolysis, leading to elevated levels of free fatty acids in the blood. These increased free fatty acids disrupt the normal structure of myocardial cell membranes, resulting in membrane instability and damage.^[12,13]^ Acidosis directly impairs myocardial function, as severe acidemia induces abnormal ion distribution across myocardial cell membranes, leading to increased intracellular calcium concentration. This results in myocardial protein hydrolysis and myocardial stunning, ultimately causing elevated troponin levels.^[4]^ Catecholamine storm and microcirculatory dysfunction: in the state of acidosis, the body experiences significant fluid loss, leading to insufficient effective circulating blood volume and resultant myocardial ischemia. Concurrently, the body is in a state of stress, with sympathetic nervous system activation and elevated catecholamine levels,^[14]^ which increase myocardial oxygen consumption, thereby exacerbating myocardial ischemia and hypoxia. Rapid correction of acidosis may induce a leftward shift in the oxygen dissociation curve, resulting in decreased oxygen release from hemoglobin and exacerbating hypoxia, which ultimately aggravates myocardial injury. Ketone bodies and inflammatory cytokines (IL-6, TNF-α) directly impair myocardial cells.^[15]^ Acidosis and glucocorticoid administration can lead to abnormal serum potassium levels,^[16]^ further causing disturbances in myocardial cellular electrical activity, elevated troponin levels, and electrocardiographic abnormalities.

Elevated thyroid hormone levels exert multifaceted effects on cardiac function,^[17]^ with hyperthyroidism demonstrating increased all-cause mortality and cardiovascular mortality. The mechanisms underlying myocardial injury in hyperthyroidism are as follows: excessive thyroid hormone levels enhance cardiac β-receptor sensitivity to catecholamines,^[18]^ while simultaneously acting on myocardial contractile proteins to augment positive inotropic effects. Thyroid hormone-mediated activation of peripheral vascular receptors induces vasodilation and reduced vascular resistance, resulting in compensatory increases in cardiac output. The elevated systemic metabolic rate in hyperthyroidism necessitates increased blood supply to meet tissue demands, with prolonged high cardiac workload ultimately leading to tachycardia and potentially progressing to heart failure and atrial fibrillation.^[19,20]^ During TS, the abrupt elevation of T3/T4 directly enhances myocardial contractility and accelerates heart rate, imposing a high-output cardiac burden. Concurrent sympathetic overactivation and massive catecholamine release may precipitate malignant arrhythmias in severe cases.^[21]^

Myocardial injury refers to the impairment of myocardial cells due to ischemia, hypoxia, inflammation, toxins, or other etiological factors, resulting in elevated levels of myocardial enzymes or troponins. This condition may not necessarily be accompanied by ischemic symptoms or dynamic electrocardiographic changes and can manifest as either transient or persistent damage.^[22]^ ST segment alterations represent a critical manifestation in electrocardiographic examinations, frequently observed in various life-threatening conditions. The following delineates the differential diagnosis of common critical illnesses associated with ST segment changes: AMI, characterized by acute coronary artery occlusion leading to myocardial ischemic necrosis, typically presents with sudden, severe, and persistent chest pain localized in the retrosternal or precordial region, often radiating to the left shoulder, medial left arm, or jaw. The pain is commonly described as crushing, oppressive, or constrictive in nature, with an intensity that is severe and duration exceeding 30 minutes, unrelieved by nitroglycerin administration. Patients frequently exhibit symptoms of restlessness, diaphoresis, fear, or a sense of impending doom, with some experiencing nausea, vomiting, arrhythmias, hypotension, shock, or heart failure. Electrocardiographic findings demonstrate ST segment elevation with upward convexity in 2 or more contiguous leads, forming a monophasic curve with the upright T wave. Within hours to days, the ST segment gradually returns to baseline, followed by T wave inversion. Concurrently, myocardial injury markers such as cardiac troponins (cTnI, cTnT) and creatine kinase-MB (CK-MB) exhibit progressive elevation following the onset of symptoms. Variant Angina: this condition is primarily caused by coronary artery spasm, often occurring in individuals with underlying coronary atherosclerosis, although it can also manifest in those with normal coronary arteries. It is characterized by episodes of chest pain that are not associated with physical exertion or emotional stress, typically occurring during rest or in the early morning hours. The pain is usually severe but short-lived, lasting approximately 10 to 20 minutes, and may be accompanied by arrhythmias during an episode. Electrocardiographic findings during an episode include ST-segment elevation in the affected leads, reciprocal ST-segment depression in the opposite leads, and tall T-waves, with rapid normalization of the ST-segment after the episode resolves. During the interictal period, the electrocardiogram may appear normal or show only T-wave changes. Cardiac biomarkers are generally within normal limits. Acute pulmonary embolism is characterized by the obstruction of the pulmonary artery and its branches by emboli, leading to pulmonary circulation impairment, which can result in increased right ventricular load. Clinical manifestations may include dyspnea, chest pain, hemoptysis, and syncope, with severe cases potentially progressing to shock. Common electrocardiographic changes include deepening of the S wave in lead I, the appearance of a Q wave and T wave inversion in lead III, as well as T wave inversion in leads V1 to V4, mild ST segment elevation or depression, and incomplete or complete right bundle branch block. D-dimer levels are typically significantly elevated, and arterial blood gas analysis may indicate hypoxemia and hypocapnia. Pulmonary computed tomography angiography is the definitive diagnostic modality. Acute pericarditis is an inflammatory condition of the pericardium that extends to the subepicardial myocardium, causing myocardial injury and resulting in ST-segment changes on the electrocardiogram. The primary symptom is chest pain, which can manifest in various forms such as sharp, crushing, or dull pain. The pain is often exacerbated by coughing, deep breathing, changes in body position, or swallowing and may radiate to the neck, shoulders, or back. Some patients may also experience fever, fatigue, and palpitations. On the electrocardiogram, ST-segment elevation with a concave upward morphology is observed in all leads except aVR and V1, accompanied by tall, upright T-waves. Over several days, the ST-segment gradually returns to normal, and the T-waves begin to invert. Subsequently, the T-waves may gradually normalize, and some patients may exhibit low QRS voltage and sinus tachycardia. Myocarditis or direct toxic injury^[23]^: the direct toxic effects of thyroid hormones and ketone bodies, or systemic inflammatory responses, lead to myocardial cell damage. Elevated concentrations of FT3 are correlated with an increased risk of coronary events. Specifically, a rise in FT3 concentration can elevate the risk of coronary events by 2.6-fold.^[24]^ Upon detection of ST segment alterations, a comprehensive analysis incorporating the patient’s clinical symptoms, medical history, and other diagnostic findings is essential to accurately identify the underlying etiology and facilitate timely therapeutic intervention. The etiology of myocardial injury in patients should also consider the potential of Takotsubo Syndrome (TTS): this condition is characterized by catecholamine storm-induced apical ballooning of the left ventricle, with its hallmark being transient and typically reversible left ventricular systolic dysfunction. The classic manifestation involves dyskinesia of the anterior septum and apex of the left ventricle, forming a “balloon-like” appearance, accompanied by compensatory hyperkinesis of the basal segments. It may also present with elevated troponin levels and ST-segment elevation. The symptoms of TTS may resemble those of a myocardial infarction, encompassing chest pain, dyspnea, and cardiac arrhythmias.^[25]^ The diagnosis of TTS typically necessitates the exclusion of other potential cardiac conditions, such as coronary artery disease, and may require pharmacological interventions including β-blockers and ACE inhibitors. The majority of patients exhibit favorable prognoses, with cardiac function generally returning to normal within days to weeks. However, this patient presented without significant clinical symptoms such as chest pain, precordial tightness, or dyspnea. Echocardiography revealed no notable abnormal myocardial motion, and ACE inhibitors were not administered during treatment. Additionally, β-blockers were discontinued once the patient’s heart rate fell below 100 beats per minute. Following the management of ketoacidosis and TS, the patient’s electrocardiogram demonstrated gradual normalization of the ST segment, and myocardial injury markers, including hsTnI and hsTnT, showed significant declines. Consequently, TTS was not considered in this case.

Based on this, we hypothesize that in the case of a patient presenting with concurrent DKA and TS: the superimposed metabolic disturbances: the hypermetabolic demands of TS exacerbate the dehydration and hyperglycemia of DKA, while the acidosis and electrolyte imbalances of DKA aggravate hyperthyroidism. During the fluid replacement therapy, the increase in effective circulating blood volume further intensifies cardiac load, exacerbating myocardial ischemia and hypoxia, ultimately leading to myocardial injury. Inflammation and oxidative stress: hyperglycemia and ketogenesis during DKA promote the release of inflammatory factors, exacerbating myocardial cell damage. The oxidative stress response in hyperthyroidism synergizes with DKA, accelerating myocardial cell apoptosis. Sympathetic nervous system and hormonal inactivation: thyroid crisis activates the sympathetic nervous system, and the high levels of catecholamines induced by the stress response during DKA further increase cardiac burden. Notably, the patient’s troponin levels began to decline after a sustained elevation, and the ST segment on the electrocardiogram gradually normalized with the correction of acidosis, both indicating an acute, reversible injury process rather than a typical myocardial infarction. In accordance with the Fourth Universal Definition of Myocardial Infarction (2018),^[26]^ despite the presence of elevated troponin levels and new ST-segment changes, the absence of typical chest pain and imaging evidence of regional wall motion abnormalities precludes the diagnosis of myocardial infarction. As highlighted in the research conducted by Liu Haifeng et al,^[27]^ DKA is associated with myocardial cell injury, which can be clinically misdiagnosed as AMI. In light of the patient’s DKA-induced metabolic disturbances and the rapid improvement of posttreatment indicators, the clinical presentation aligns more closely with the diagnostic category of “type 2 myocardial infarction (T2MI)” or “acute myocardial injury” secondary to noncoronary artery occlusion. This condition is characterized by myocardial damage resulting from an imbalance between oxygen supply and demand, rather than AMI. However, definitive exclusion of coronary artery pathology requires subsequent coronary PCI. Due to the patient’s uncontrolled hyperthyroidism, coronary angiography has not yet been performed, precluding definitive confirmation or exclusion of type 2 myocardial infarction diagnosis. This represents a limitation in the current case analysis. However, precisely due to these limitations, the existing diagnostic and therapeutic approaches in this case provide valuable insights for readers, prompting us to adopt a more comprehensive perspective in clinical practice when evaluating patient conditions. Although this patient received treatment strictly in accordance with the DKA and TS guidelines, severe myocardial injury still occurred on the third day of treatment. Analysis revealed that the underlying mechanism involved increased effective circulating blood volume, elevated cardiac preload, and exacerbated myocardial ischemia and hypoxia, leading to cardiomyocyte damage. This was driven by a metabolic-inflammatory cascade reaction jointly induced by DKA and TS, with mechanisms encompassing hemodynamic changes, direct toxic effects of acidosis, and catecholamine-mediated microcirculatory dysfunction. Early identification of such myocardial injuries requires close integration with clinical context, avoiding overreliance on traditional diagnostic frameworks for myocardial infarction.

Myocardial injury in DKA complicated with TS results from the combined effects of metabolic disorders, inflammatory responses, sympathetic storm, and cardiac overload. The treatment strategy necessitates simultaneous correction of DKA and control of TS, active cardiac function protection, and multidisciplinary collaboration when indicated. Dynamic monitoring, early recognition, and comprehensive intervention are crucial for life-saving and prognosis improvement. Current clinical management predominantly relies on empirical treatment. In the era of vascular intervention and thrombolytic therapy, this approach may expose patients to unnecessary medical procedures with significant complication risks. Future research should focus on integrating basic and clinical studies to explore effective early warning indicators for timely intervention, thereby improving outcomes in this patient population.

## Author contributions

**Investigation:** Yingjuan Ma, Ruirui Liu, Xinyue Li.

**Resources:** Yingjuan Ma, Ruirui Liu.

**Supervision:** Limin Jia.

**Visualization:** Ting Bai, Wang Chang.

**Writing – original draft:** Yingjuan Ma, Ruirui Liu.

**Writing – review & editing:** Limin Jia.
